# Targeting Spinal Interneurons for Respiratory Recovery After Spinal Cord Injury

**DOI:** 10.3390/cells14040288

**Published:** 2025-02-15

**Authors:** Maha Paracha, Allison N. Brezinski, Rhea Singh, Elizabeth Sinson, Kajana Satkunendrarajah

**Affiliations:** 1 Department of Neurosurgery, Medical College of Wisconsin, Milwaukee, WI 53226, USA; mparacha@mcw.edu (M.P.); abrezinski@mcw.edu (A.N.B.); rhea.singh219@gmail.com (R.S.); beth.sinson@gmail.com (E.S.); 2 Clement J. Zablocki Veterans Affairs Medical Center, Milwaukee, WI 53295, USA; 3 Department of Physiology, Medical College of Wisconsin, Milwaukee, WI 53226, USA

**Keywords:** breathing, interneurons, neuroplasticity, spinal cord injury

## Abstract

Spinal interneurons (SpINs) are pivotal to the function of neural circuits, orchestrating motor, sensory, and autonomic functions in the healthy, intact central nervous system. These interneurons (INs) are heterogeneous, with diverse types contributing to various neural systems, including those that control respiratory function. Research in the last few decades has highlighted the complex involvement of SpINs in modulating motor control. SpINs also partake in motor plasticity by aiding in adapting and rewiring neural circuits in response to injury or disease. This plasticity is crucial in the context of spinal cord injury (SCI), where damage often leads to severe and long-term breathing deficits. Such deficits are a leading cause of morbidity and mortality in individuals with SCI, emphasizing the need for effective interventions. This review will focus on SpIN circuits involved in the modulation of breathing and explore current and emerging approaches that leverage SpINs as therapeutic targets to promote respiratory recovery following SCI.

## 1. Introduction

Respiratory impairment is the leading cause of morbidity and mortality in SCI, impacting over 300,000 individuals in the United States [1]. Respiratory neural networks are organized across the cervical, thoracic, and lumbar spinal cord levels, with descending respiratory drive originating from brainstem respiratory nuclei [2,3,4,5,6,7,8]. The severity and location of the SCI significantly influence the respiratory impairment that ensues. With over 60% of injuries occurring at the cervical level [9], high cervical injuries disrupt the connectivity between supraspinal respiratory networks and the diaphragm—the primary muscle for inspiration—resulting in significant respiratory dysfunction [10]. SCI can be classified as complete or incomplete, with the majority being incomplete. In these cases, some respiratory spinal neural circuitry remains preserved. Complete SCI results in a total loss of sensorimotor and anatomical function, leading to a much more severe respiratory impairment, characterized by reduced tidal volume, shallow breathing, and weak phrenic nerve bursting [11]. In contrast, following incomplete SCI, there are varying levels of spontaneous recovery of breathing, reflecting the spared respiratory neural circuitry and potential for therapeutic intervention. Developing treatments to improve breathing after SCI requires a foundational understanding of the molecular, physiological, and anatomical mechanisms that drive respiratory circuits.

Research on spinal neural circuits began in 1906, when Charles Sherrington first characterized spinal reflexes and their underlying mechanism [12]. Following this milestone, several pioneering studies have expanded our understanding of spinal circuits that regulate motor function. One such noteworthy study involved generating spontaneous respiratory rhythms in high-spinalized cats [13]. Aoki et al. (1980) conducted a series of experiments involving electromyography (EMG) to monitor the diaphragm and intercostal muscles in these spinalized cats. The findings revealed that neurons at the C1 level of the spinal cord can generate spontaneous rhythmic breathing independent of supraspinal input; however, this spinal respiratory activity was insufficient to maintain rhythmicity over extended periods without the descending inputs from the medullary respiratory center [13]. This research was a critical step in furthering our understanding of respiratory regulation at the spinal cord level. Subsequent studies built upon this knowledge. By employing optical imaging with a voltage-sensitive dye, Oku et al. (2008) revealed a respiratory-related region in the high cervical spinal cord that modulates the activity of intercostal and phrenic nerves [14].

While significant strides have been made in our understanding of the neural control of breathing, neuroplasticity in the spinal perilesional tissue remains a topic that is still obscure. Spinal neuroplasticity enables neurons within a circuit to undergo structural and functional adaptations, which results in changes to sensorimotor function. This plasticity includes alterations in synaptic strength, the formation of new synapses, and the activation of previously inactive circuits [15]. These adaptive responses are important for the nervous system’s ability to respond to short-term and long-term disorders, especially following SCI.

The crossed phrenic phenomenon (CPP) is the most illustrated form of respiratory neural plasticity following SCI, wherein the function of the ipsilateral diaphragm can recover partially via spared connections following a C2 hemisection injury (C2Hx) [16,17]. This recovery can occur spontaneously over weeks or immediately after a contralateral phrenectomy, indicating the presence of a latent or dormant circuit [18,19,20]. Goshgarian (2003) suggests that this return of activity is due to the increased signal strength from the remaining synapses in a monosynaptic pathway [16]. Other studies have used electrical recordings to show the presence of a polysynaptic pathway along with monosynaptic pathways that are responsible for the CPP [18]. Ling et al. (1995) electrically stimulated the contralateral spinal cord and recorded from the ipsilateral phrenic nerve [21]. Using these recordings, they showed that the phrenic nerve responded with short and long latencies indicative of polysynaptic connections [21]. Transsynaptic labeling techniques have identified SpINs as crucial components of the polysynaptic network [17,22,23]. Using a pseudorabies virus (PRV) for retrograde transneuronal tracing, researchers have observed a significant increase in PRV-labeled INs ipsilateral to the C2Hx, demonstrating robust recruitment of excitatory INs in the two weeks following SCI [23]. In summary, the CPP serves as a key example of respiratory neuroplasticity, demonstrating how monosynaptic and polysynaptic pathways, including SpINs, contribute to the recovery of diaphragm function after SCI. Greater knowledge of these SpINs within the respiratory circuit in health and after SCI is essential to develop effective therapeutics for SCI-induced respiratory dysfunctions.

This review will explore the neural networks involved in modulating breathing, primarily focusing on SpIN circuits, with some mention of their inputs from brainstem INs and output through spinal motor neurons. It will also examine the effects of SCI on these circuits and highlight current preclinical studies targeting these IN networks to improve respiratory recovery.

## 2. Respiratory Dysfunction After Spinal Cord Injury

SCIs can have profound implications for respiratory function, as the spinal cord plays a crucial role in regulating the muscles responsible for breathing. The diaphragm, which is the primary muscle for inspiration, is innervated by phrenic motor neurons (PMNs) located in cervical spinal levels C3–C6 [24]. Consequently, patients with injuries at or above the C5 level often require ventilatory support due to impaired diaphragmatic function [25,26]. Individuals with injuries below the C5 level may not necessarily need respiratory support, but they often experience various degrees of breathing dysfunction, partly due to impairments in the accessory breathing muscles. Patients with long-term respiratory insufficiencies are at a higher risk of experiencing low lung volumes, weak coughing, pulmonary edema, sleep-disordered breathing, and cardiovascular issues [27]. Breathing complications, such as atelectasis, pneumonia, and ventilatory failure, are leading causes of morbidity and mortality following SCI [25,28]. These complications can develop rapidly, affecting 36–83% of patients, with some issues potentially becoming permanent [25]. The severity and location of the SCI significantly influence the extent of these dysfunctions [27].

Respiratory function requires the precise coordination of multiple muscle groups involved in breathing. Motor neurons that innervate these muscles are in different segments of the spinal cord. The neural inputs transmitted to muscle groups involved in respiratory function can change after SCI due to the mechanical disruption of descending respiratory drive from brainstem networks as well as a direct impact on spinal respiratory neurons. The level of injury determines which neural networks responsible for controlling and coordinating these respiratory muscles are affected [29]. When these muscles act independently or unequally, irregular chest movement disrupts airflow [30]. A clear understanding of the spinal mechanisms underlying the neural control of breathing is essential to explain why the level of injury critically influences respiratory function and patient outcomes.

## 3. Neural Control of Breathing

### 3.1. Spinal Control of Respiratory Muscles

In healthy conditions, the diaphragm is the primary respiratory muscle during inspiration, while expiration is mostly a passive process [31,32]. However, accessory muscles, including those in the upper airways, intercostals, abdominals, pectoralis major, scalenes, and sternocleidomastoids, play integral roles in supporting inspiration and other ventilatory functions [31].

The intercostal muscles, innervated by motor neurons in the thoracic spinal cord (T1–T11), assist during inspiration by pulling the rib cage upward and outward [32]. These muscles are particularly significant in individuals with cervical spinal cord injury (cSCI), where the diaphragm paralysis necessitates additional muscular support to maintain breathing [25]. The scalene and sternocleidomastoid muscles stabilize the rib cage during inspiration and are innervated by motor neurons located at spinal levels C4–C8 and brainstem to C2, respectively [28,31].

Abdominal muscles contribute to both inspiration and expiration. Before inspiration, these muscles store elastic energy in the chest wall, enhancing the diaphragm’s inspiratory force [31]. During forced expiration, abdominal muscles’ contractions expel air from the lungs while the chest wall muscles relax [30]. These muscles are innervated by motor neurons spanning the thoracic and lumbar spinal cord (T6-L3). Injury to this region can impair the cough reflex, increasing the risk of aspiration pneumonia.

**Figure 1:**
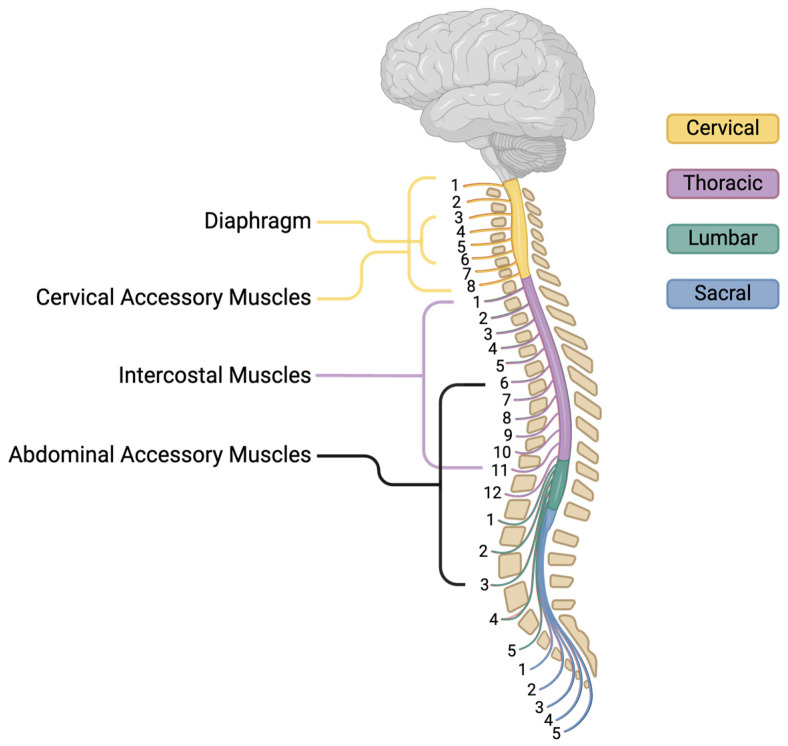
Respiratory muscles and their corresponding spinal levels.

Figure 1 summarizes the respiratory muscles and their spinal innervation. When visualizing the system, it becomes obvious how cSCI disrupts the coordination between the diaphragm, intercostals, and cervical and abdominal respiratory muscles, impairing respiratory efficiency. Effective management of the respiratory function in these individuals requires interventions that support ventilation, maintain airway patency, and reduce the risk of respiratory complications.

### 3.2. Supraspinal Modulation of Breathing

The typical breathing pattern involves inspiration, passive expiration, and, in some cases, a post-inspiratory phase. The regulation of respiratory muscles responsible for this pattern is controlled by descending input from respiratory nuclei within the brainstem [32]. Central pattern generators (CPGs), neural circuits in the brainstem, autonomously sustain rhythmic breathing while dynamically adjusting it to meet physiological demands [32]. Table 1 summarizes the roles of various nuclei in generating and modulating these respiratory patterns.

Respiratory rhythm generation and pattern formation are attributed to several regions in the ventrolateral medulla, including the pre-BötC, the BötC, the pFRG, and the PiCo [33,34]. Inspiratory rhythm primarily originates from the pre-BötC, where glutamatergic INs form a recurrently connected network that generates the rhythmic bursts required for breathing. The excitatory INs in the pre-BötC fire after a certain refractory period, while the inhibitory INs modulate the firing frequency of the excitatory INs [35,36,37,38,39,40].

**Figure 2:**
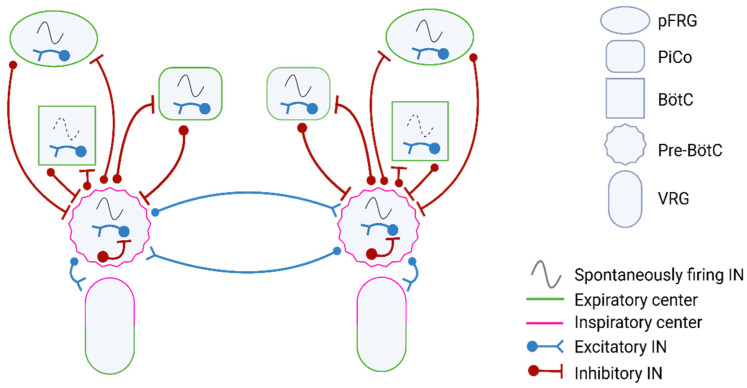
Respiratory rhythm and pattern generating IN networks in the brainstem.

The excitatory INs of the pre-BötC have key connections within the respiratory network to regulate breathing. They transmit excitatory signals to the spinal respiratory motor nuclei via the rVRG, driving inspiratory muscle activity. Additionally, they connect to the pFRG through inhibitory INs, helping regulate expiratory activity. The pre-BötC also interacts with the BötC, which sends back inhibitory signals to modulate and refine the respiratory rhythm [35,41,42]. While Bötc is not rhythmogenic in adulthood under normal conditions, evidence suggests that glycinergic Bötc INs can generate an expiratory rhythm during hypoxia in vivo or contribute to post-inspiratory control [43]. However, post-inspiration is primarily regulated by PiCo, which is rhythmogenic and consists of a heterogeneous population of INs capable of eliciting post-inspiratory bursts in vivo [32,43,44,45,46]. Meanwhile, pFRG activity drives active expiration but remains mostly inhibited at rest [32,35,47,48]. Figure 2 illustrates the respiratory rhythm and pattern-generating IN networks in the brainstem.

Beyond maintaining baseline respiratory function, CPGs integrate inputs from chemoreceptors that monitor CO_2_ and O_2_ levels, sensory feedback from lung mechanoreceptors, and higher-order cortical signals that facilitate voluntary breath control [32,48,49]. This integration allows the respiratory CPGs to regulate the breathing rate and depth in response to various physiological demands, such as exercise, speech, and emotional stress [48]. The adaptability of the brainstem respiratory network ensures efficient ventilation, even under chronic physiological changes, including respiratory disease, SCI, or altitude adaptation [49]. Ultimately, brainstem respiratory CPGs function as dynamic regulatory hubs, synchronizing and modulating breathing to maintain respiratory homeostasis under diverse conditions.

### 3.3. Premotor Bulbospinal Control of Spinal Respiratory Networks

Premotor bulbospinal neurons in the rVRG and cVRG transmit inspiratory and expiratory rhythms to PMNs within the cervical spinal cord [33]. Respiratory motor neurons generate breathing patterns by receiving excitatory inputs from these descending projections [50,51]. The rVRG connects to PMNs in the phrenic nucleus, which innervates the diaphragm, driving inspiration [24].

IN networks form relays between bulbospinal neurons and spinal respiratory motor neurons within the spinal cord. These SpIN networks play an integral role in modulating respiratory output, particularly after SCI, where they compensate for disrupted inputs from the brainstem [52,53]. The importance of SpINs in respiratory adaptation underscores their role in neural plasticity and recovery after SCI. Given the extensive research on the neural control of breathing, numerous comprehensive reviews exist on this topic [16,54,55]. Therefore, this review will focus on the specific contributions of SpIN networks to respiratory control, particularly in the context of SCI-related respiratory dysfunction.

## 4. Spinal Interneurons: Classification and Functional Importance

SpINs are multifunctional neurons integral to spinal cord function. They exert influence across the spinal cord through long-range ascending and descending projections or modulate neural activity locally within the same spinal segment. This modulation occurs either ipsilaterally or contralaterally via commissural neurons. Recent advancements in genetic and molecular neuroscience have significantly enabled the ability to classify and characterize SpINs, revealing their vast heterogeneity. Broadly, they are categorized into two cardinal classes distinguished by their anatomical location in the spinal cord laminae: dorsally located SpINs, primarily involved in somatosensory processing, and ventrally located SpINs, mainly associated with motor control. While this classification may be an oversimplification, it forms the foundation for further subdivisions by developmental origins, transcriptomic profiles, and electrophysiological properties, with populations broadly categorized as excitatory or inhibitory. Extensive summaries of IN classes have been previously provided [56,57,58]. However, we provide brief summary tables for the reader’s convenience (Table 2 and Table 3).

Our current understanding of SpINs has been largely shaped by research focused on locomotor circuits and CPGs [56,67,71,72,73,74]. However, emerging evidence implicates ventrally derived SpIN in respiratory motor control, with significant contributions to neuroplasticity and recovery after SCI [19,20,23,33,54,75,76,77,78,79,80,81]. Due to the motor control roles of ventral SpIN populations and their demonstrated importance in respiratory regulation and SCI recovery, this review predominantly focuses on ventral SpIN populations. Still, it would be remiss to disregard dorsally derived neurons; as such, dorsal SpINs and some of their key functions are briefly summarized in Table 3.

### 4.1. Ventral Spinal Interneurons in Motor Function

Ventrally located SpINs, comprising both excitatory and inhibitory subclasses, are pivotal in shaping motor function [33,56,67,69,70,71,82,83,84]. Excitatory INs participate in the initiation and transmission of action potentials, promoting coordinated muscle activation and movement. Conversely, inhibitory populations temper excessive neuronal activity by mitigating excessive excitation, thereby preventing hyperactive responses that could lead to uncoordinated or unintended muscle contractions. This delicate interplay between excitatory and inhibitory signals ensures the precise regulation of motor output, allowing for smooth and coordinated movements throughout the body.

A greater understanding of these spinal networks, particularly those controlling breathing, becomes especially vital in the context of neurological disorders such as amyotrophic lateral sclerosis (ALS) and SCI, where respiratory function is often significantly impacted. Consequently, this section delves into the role of SpINs in the control of breathing, specifically in the context of their importance in addressing respiratory complications associated with neurological injury.

### 4.2. Spinal Interneurons in the Neural Control of Breathing

Early studies of spinal respiratory networks identified distinct populations of SpINs associated with the inspiratory, expiratory, and post-inspiratory phases of breathing [85]. Electrophysiological investigations into bulbospinal respiratory neurons demonstrated monosynaptic input to upper cervical respiratory neurons in rats, highlighting a direct connection between the brainstem and the spinal cord [4]. This connection was later supported by anterograde viral tracing studies, confirming that neurons from the rVRG project directly to cervical SpINs, establishing that SpINs receive direct input from respiratory brainstem centers. Importantly, Duffin and Douse were among the first to describe direct monosynaptic connections between cervical SpINs and PMNs using antidromic mapping and spike-triggered averaging to monitor SpIN firing during respiration [13,86]. Despite advancements in circuit-mapping technologies, including viral tracing and electrophysiology, the full extent of respiratory brainstem SpIN networks is not known and requires further investigation.

Interestingly, studies have shown that SpINs could function independently without supraspinal input [22,80,87,88]. Experiments on cats spinalized at the C1 level demonstrated the intrinsic capacity of the spinal cord to generate spontaneous respiratory rhythms. This finding was pivotal in implicating the spinal cord as a potential site for rhythm and pattern generation [13]. In a pivotal study by Cregg et al. (2017), researchers identified a spinal network of excitatory SpINs that were both sufficient and necessary to drive PMN burst activity in the absence of bulbospinal input [80]. This study showed that optogenetic stimulation of PMNs alone was insufficient to elicit PMN bursts, indicating that phrenic motor bursting is reliant on premotor excitatory input from spinal networks. Additionally, in experiments involving C2Hx, which disrupts bulbospinal input from the brainstem respiratory centers, the application of the disinhibitory agents picrotoxin (PTX) and strychnine (STRYCH) induced PMN bursts and restored diaphragm activity in previously paralyzed muscle [80]. These findings suggest that excitatory spinal networks can work independently and generate respiratory motor activity without brainstem input centers but are regulated by inhibitory synaptic inputs to modulate diaphragmatic motor function effectively. This capability highlights the potential for spinal networks to compensate for lost supraspinal control in conditions such as SCI.

### 4.3. Excitatory V2a Interneurons in Respiratory Control

In line with this, research into the role of excitatory V2a INs in breathing, particularly in the context of ALS, has yielded critical insights into spinal respiratory circuits. Genetic ablation of V2a INs in neonatal mice leads to irregularity in breathing patterns, decreased respiratory frequency, and often death [89]. This dysfunction becomes more exacerbated in the later stages of ALS, where V2a neurons are preferentially lost [90]. However, it appears the phenotype is not solely due to the loss of V2a INs themselves but, rather, the lack of excitatory drive to the pre-BötC because respiratory irregularities could be mitigated by the addition of substance P or *N*-methyl-d-aspartate (NMDA), which enhance respiratory regularity and the frequency of respiratory rhythms. Interestingly, chemogenetic silencing of V2a INs in adult mice did not dramatically affect breathing rhythm or diaphragm function, suggesting that the role of V2a INs evolves with age. While V2a neurons are critical for respiratory rhythm generation in neonatal mice, their primary function in adults appears to shift toward patterning accessory respiratory muscles rather than directly driving diaphragm contractions [33]. This developmental shift does not imply redundancy in diaphragm control during adulthood; instead, V2a INs are likely contributing to alternative respiratory demands, such as those during exercise, where enhanced inspiratory drive is required. These findings underscore the dynamic role of V2a neurons across life stages and their adaptability in supporting respiratory function under varying physiological demands.

## 5. Spinal Interneurons in Respiratory Recovery After Spinal Cord Injury

Recent findings emphasize the critical role of cervical excitatory SpINs in respiratory recovery following SCI. Satkunendrarajah et al. (2018) demonstrated that while cervical excitatory INs are not essential for eupneic breathing in healthy animals, they become necessary for maintaining breathing after non-traumatic and traumatic SCI. This study also showed that chemogenetic stimulation of a small population of excitatory SpINs in the intermediate gray matter at the C4–C5 spinal levels is sufficient to promote respiratory recovery following injury [79]. These findings were later supported by Jensen et al. (2024), who reported that systemic chemogenetic stimulation of both bulbospinal and spinal V2a neurons enhanced respiratory-related diaphragm activity two weeks after C2Hx [91].

Furthermore, studies have revealed that an increased number of V2a INs are anatomically incorporated into the spinal respiratory network within two weeks after cSCI, suggesting compensatory recruitment to sustain respiratory function. This recruitment may facilitate the formation of new synaptic pathways, potentially restoring connectivity between supraspinal neurons and spinal segments below the lesion. Notably, these observations indicate that these INs can reroute descending neural signals around a spinal lesion, aiding motor recovery after a C2Hx [92]. Streeter et al. (2019) demonstrated this after C2Hx, ipsilateral cervical SpINs, with synaptic information flowing directionally from the contralateral to the ipsilateral side [19]. Collectively, these findings underscore the role of cervical SpINs in neuroplasticity and recovery. Together, these studies support the idea that while V2a neurons may not be essential for eupneic breathing, they are likely recruited and then utilized to sustain breathing after damage to the respiratory network [23].

The regeneration of neurons and their projections through severe SCI is particularly challenging; however, functional recovery seems to depend more on the reorganization and adaption of specific neuronal subpopulations than on the regeneration of cells within the injury site [79,93]. For instance, the involvement of developmentally defined V2a neurons in the recovery of walking and breathing indicates that these cells alter their function within the circuits responsible for these functions [23,74,91]. Similarly, dorsally derived INs, such as the dl3 subtype, have also been implicated in functional adaptation and recovery after SCI [94,95], suggesting that recovery relies on the capacity of specific neuronal subtypes to adapt and assume roles within the newly formed circuits.

### 5.1. Inhibitory Spinal Interneurons in Respiratory Regulation

While much of the work surrounding SpINs and breathing has primarily centered around excitatory INs, it is important to recognize that inhibitory INs also have a critical role in the regulation of breathing. Electrophysiological studies from Duffin et al. (1986) found direct monosynaptic connections between inhibitory SpINs and ipsilateral PMNs [86]. These findings were later supported by Streeter et al. (2017), who demonstrated that cervical inhibitory SpINs also project to contralateral PMNs [20]. In both cases, these inhibitory SpINs were found to be primarily active during expiration, functioning to constrain the excitation of PMNs during this passive respiratory phase. Additional studies investigating inhibitory SpINs further highlighted the importance of inhibitory neuronal mechanisms in respiratory control. For example, blocking GABA-A receptors in the spinal cord can significantly increase peak phrenic nerve amplitude and induce crossed phrenic activity after SCI, restoring activity in previously silent phrenic nerves [96]. Similarly, antagonism of GABA-A and glycine receptors and consequent disinhibition at the site of C1–C2 pre-phrenic INs can induce activity in phrenic nerves and initiate bilaterally coordinated phrenic bursting [18,80]. Consequently, this demonstrates that inhibitory neurons modulate the activity of phrenic nerves. These findings underscore the modulatory role of inhibitory SpINs in maintaining balanced respiratory motor output and enabling compensatory mechanisms following SCI. However, much remains unknown about respiratory-related inhibitory SpINs, including IN subclasses and the full extent of their connections in the brainstem and spinal respiratory networks.

### 5.2. Spinal Interneurons in Hypoxic Responses

SpINs also play a dynamic role in adaptive responses to respiratory challenges, such as hypoxia and hypercapnia. Sandhu et al. (2015) identified distinct populations of mid-cervical SpINs that respond differentially to hypoxia using multielectrode arrays. They identified three primary types of firing patterns in mid-cervical SpINs based on their responses to hypoxia: neurons activated by hypoxia, inhibited by hypoxia, and sensitive to hypoxia [77]. Interestingly, over 80% of these cells altered their bursting activity during hypoxia, and 40% continued to exhibit altered firing patterns even after hypoxia ended [77]. Further, bouts of acute intermittent hypoxia (AIH) resulted in reduced inhibitory input and increased excitatory input to PMNs, leading to heightened activity during hypoxia compared to baseline levels. This indicates robust recruitment of neurons to adaptively respond to low oxygen levels for the hypoxic response [20].

Interestingly, studies of chronic SCI reveal that the hypoxic responses of SpINs vary depending on their location relative to the lesion (ipsilateral vs. contralateral), with intermittent hypoxia enhancing cervical SpIN connectivity [20]. The mechanisms behind SpIN response to hypoxia remain unclear. However, recent evidence suggests that neurons in the spinal intermediolateral nucleus can independently sense oxygen levels without input from the bulbospinal pathways or carotid body [97]. This raises the intriguing possibility that cervical spinal neurons possess intrinsic oxygen-sensing capabilities or rely on alternative mechanisms to modulate activity during hypoxia.

## 6. Therapeutic Interventions After SCI: The Importance of Intervention Timing

In SCI recovery, early intervention is widely regarded as critical for achieving the best possible patient outcomes, often summarized by the phrase “Time is Spine” [98]. As secondary injury progresses, factors such as glial scarring, neuronal loss, and tissue degeneration further exacerbate damage, making neurological recovery increasingly difficult. Given the limited regenerative capacity of the adult spinal cord, axon regeneration and neurogenesis are not well documented, particularly within or across the lesion site [99]. Studies suggest that early intervention is ideal and leads to the most significant improvements. One study found that the odds of a 2 or greater AIS score improvement were over two-times more likely in individuals with early vs. late surgical interventions [100]. While early intervention is best, interventions in the acute phase are not always feasible for promoting plasticity due to several key factors. During the acute phase (hours to days post-injury), the spinal cord undergoes secondary injury processes, including neuroinflammation, excitotoxicity, ischemia, and cell death, creating an unstable environment that hinders neural repair and plasticity. Additionally, the nervous system is still undergoing rapid degeneration and structural changes, preventing neurons from establishing the stable synaptic connections necessary for functional recovery. While early interventions can help minimize damage, they may also disrupt the natural progression of glial scar formation and wound stabilization, both of which impact neuroprotection and plasticity. Furthermore, neuroplasticity mechanisms typically emerge in the subacute and chronic phases, when spared pathways begin to reorganize and compensate for lost function. Early neuromodulatory interventions could also risk maladaptive plasticity, leading to issues such as spasticity or neuropathic pain. From a clinical standpoint, acute interventions are often logistically challenging, as patient stabilization, diagnosis, and access to specialized care can delay treatment. While interventions during the acute phase aim to limit damage and preserve function, true circuit reorganization and plasticity-driven recovery often require the more stable neural environment of the subacute or chronic phase. This underscores the importance of timing-dependent strategies when developing therapeutic interventions for promoting neuroplasticity via spared circuits.

**Figure 3:**
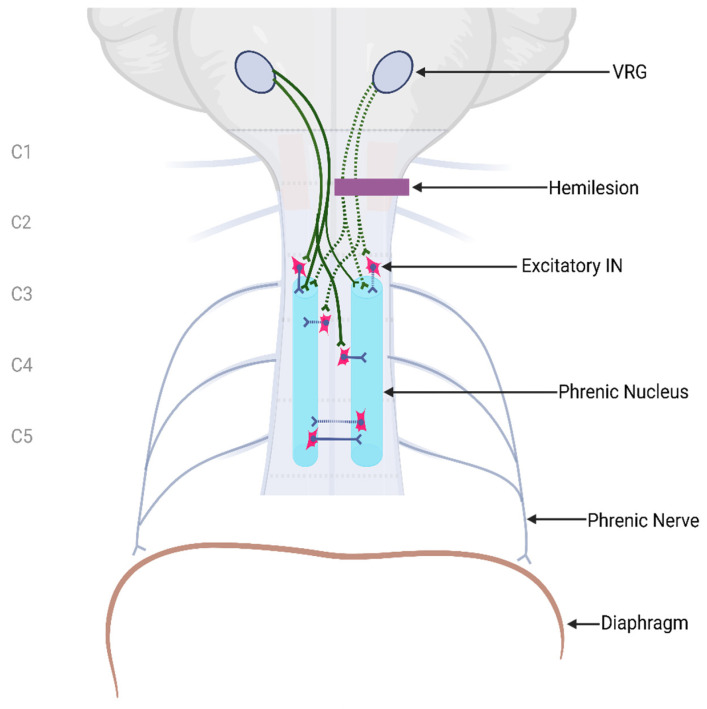
Excitatory interneuronal pathways activated after a high cervical hemisection.

Our knowledge of how spared, latent, and newly formed circuits integrate at different time points following injury is essential for developing novel therapeutic approaches. Some studies demonstrate that latent pathways become active almost immediately following traumatic cSCI, while regeneration and spontaneous recovery may occur weeks to months after injury in mice [101,102]. A striking example of time-dependent plasticity and latent pathway activation is observed in CPP. In early studies on guinea pig respiratory circuits, immediate contralateral phrenectomy following C2Hx failed to induce the CPP, leading to asphyxiation [103]. However, subsequent research by Goshgarian and Guth found that the CPP could be induced as early as 3.5 h after C2Hx with contralateral phrenectomy, suggesting the presence of latent synapses that require time to become active following injury [104]. The Goshgarian group later demonstrated that rat pups exhibited spontaneous CPP activity as early as post-natal day 2, without requiring contralateral phrenectomy. This spontaneous CPP can be elicited until post-natal day 28 but gradually declined, becoming fully latent by day 35 [105]. Further studies examining CPP in rats found that young rats needed between 1 and 7 days post-SCI for the CPP to be active, while in older rats, the CPP was induced immediately after injury [106]. Later studies found that the CPP was spontaneously activated in young rats at 2 weeks, while others observed activation at 4–6 weeks post-injury [107]. These studies perfectly demonstrate the variation in time- and age-dependent alterations to neural networks, which may also be influenced by strain differences. Figure 3 illustrates the excitatory IN pathways that can be activated after a high hemisection cSCI. 

During injury, physical remodeling is accompanied by changes to complex neuronal signaling cascades, and our understanding of the molecular mechanisms underlying SpINs’ role in post-injury plasticity remains incomplete. While serotonin signaling has been widely implicated as a key driver of recovery following cSCI, it is neither the sole mechanism nor exclusive of SpINs. Recovery likely involves multiple molecular pathways, necessitating further research to elucidate these additional contributing factors. A comprehensive exploration of the molecular basis of SCI-related plasticity is beyond the scope of this review; however, this is a critical avenue for ongoing research. Future investigations are necessary to determine the precise functional, anatomical, and neurochemical aspects of how circuits change at various stages post-injury.

## 7. Therapeutic Targeting of Spinal Interneurons After SCI

**Figure 4:**
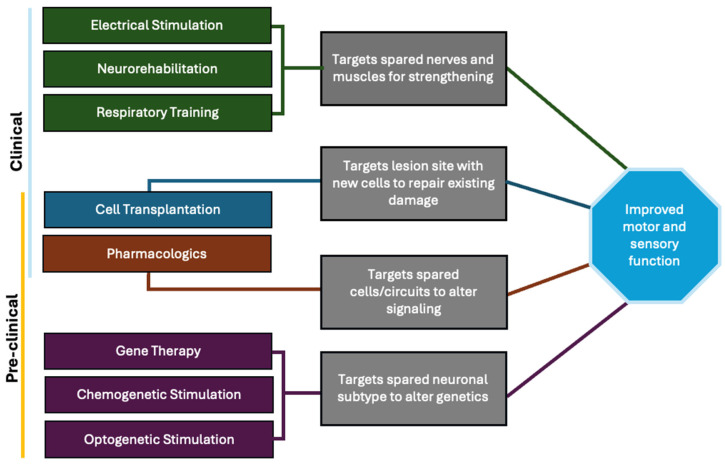
Graphical summary of current preclinical and clinical interventions and corresponding targets.

Research into SCI treatments focuses on multiple approaches, including restoring damaged pathways, rewiring dormant connections, and creating new neural circuits. In addition to directly targeting neuronal connections, indirect approaches aimed at modulating glial activity are also being investigated. Since SpINs exhibit remarkable plasticity, they represent an appealing target for interventions that aim to restore respiratory and motor functions post-injury. Below, we discuss the key therapeutic possibilities aimed at enhancing SpIN-mediated neuroplasticity, and Figure 4 shows a graphical summary of the current preclinical and clinical interventions utilizing SpINs.

### 7.1. Electrical Stimulation of Spinal Interneurons

Targeted spinal stimulation is an emerging strategy to promote functional recovery after SCI. Indeed, numerous studies have reported significant improvements in hand and walking ability across various stages of injury [108,109]. A pivotal study utilizing single-cell recovery atlas analysis revealed that a subpopulation of excitatory SpINs expressing Vsx2, previously considered non-essential for walking, became indispensable for locomotor recovery when combined with epidural electrical stimulation [74]. Despite these advances in locomotor rehabilitation, the role of SpIN-targeted electrical stimulation in respiratory recovery is underexplored.

DiMarco and Kowalski have conducted extensive research on epidural spinal stimulation for respiratory recovery and support. In 2005, they demonstrated that combining intercostal and diaphragm pacing could effectively sustain ventilatory support in tetraplegic patients [110]. By 2009, they attributed this effect to the activation of spinal cord tracts that synapse with the inspiratory motor neuron pools, namely the INs [111]. However, further investigation revealed that INs are not essential for this response. Instead, neuronal tracts in the lateral funiculus can either activate INs or directly synapse with the PMNs, thereby eliciting respiratory activity [112].

In an initial direct stimulation study, Sunshine et al. (2018) applied microstimulation via implants spanning the spinal cord from the C2 to T1 levels. This approach successfully activated the diaphragm with longer latencies, indicating the recruitment of polysynaptic circuits mediated by SpINs [113].

Indirect electrical stimulation techniques, such as the activation of phrenic proprioceptive or nociceptive afferents, have shown potential in engaging respiratory SpINs to enhance function. Razook et al. (1995) demonstrated that stimulating nociceptive phrenic afferents effectively activated SpINs within the upper cervical segments, while Malakhova and Davenport (2001) found that stimulating small-diameter nociceptive afferents in the phrenic nerve triggered neuronal responses within the C3–C5 dorsal horn [114,115]. These dorsal horn neurons are implicated in respiratory reflexes, suggesting that indirect activation of sensory pathways could modulate the IN circuits essential for breathing [55].

The integration of closed-loop stimulation systems offers a significant advancement in respiratory rehabilitation by precisely targeting key IN circuits, thereby enhancing neuroplasticity and optimizing alternate pathways to restore respiratory function following SCI [116]. In contrast, continuous epidural electrical stimulation has demonstrated limited effectiveness in locomotor recovery in individuals with SCI, primarily due to the out-of-phase activation of inhibitory INs, which disrupts motor function [117]. Clinical evidence further underscores the superiority of closed-loop stimulation. A study involving three SCI patients revealed that closed-loop electrical stimulation significantly outperformed continuous stimulation [118]. Building upon this approach, Dale lab (2022) applied closed-loop stimulation in a C2Hx SCI model, observing that just three days of closed-loop electrical stimulation lowered the motor threshold. This finding suggests that previously low-responsive or non-responsive components of the phrenic motor network became excitable. This elevated excitability facilitated the emergence of late responses, which the researchers attributed to heightened activity within phrenic-related IN populations [119].

These findings highlight the untapped potential of electrical stimulation techniques in promoting both locomotor and respiratory recovery through the targeted engagement of SpINs. Currently, diaphragm pacing is available as an effective method for providing respiratory support in ventilator-dependent patients with cSCI. However, its success is limited, benefiting only half of the patients [112,120]. Alternative approaches that stimulate intercostal and phrenic muscles via SpINs may offer additional respiratory support for patients who are unsuitable for diaphragm pacing. However, additional animal and clinical studies are necessary to establish the feasibility and effectiveness of these techniques in the SCI population [112].

### 7.2. Chemogenetic and Optogenetic Stimulation

Advancements in genetics and molecular biology have revolutionized the development of highly selective neuromodulation techniques, with chemogenetic and optogenetic approaches emerging as pivotal tools for investigating SpINs and their role in motor control and recovery following SCI. Chemogenetic stimulation, particularly through Designer Receptors Exclusively Activated by Designer Drugs (DREADDs), allows for precise modulation of neuronal activity via engineered G-protein coupled receptors (GPCRs) that respond selectively to synthetic ligands. These GPCRs can be genetically expressed or delivered via viral injection to a targeted neuronal subpopulation [121,122]. For controlled modulation, a ligand can be administered intraperitoneally for short-term effects or provided orally through drinking water for long-term modulation [122,123]. Clozapine-N-Oxide (CNO) is one of the most commonly used ligands; however, it undergoes partial conversion to Clozapine, which can interfere with respiratory responses to hypercapnia [122,123,124]. To mitigate these effects, small doses of CNO or alternative ligands, such as Compound **21**, should be considered for respiration-related studies [122,123]. These advances in chemogenetics pave the way for more targeted and effective neuromodulatory interventions in SCI rehabilitation.

Using chemogenetics, studies have begun selectively inhibiting or activating SpINs to understand their function and integration in various systems. By selectively expressing DREADDs on excitatory SpINs in the cervical spinal cord, Satkunendrarajah et al. (2018) found that stimulating these neurons immediately after traumatic SCI restores function to the previously paralyzed hemi-diaphragm [79]. Additionally, research by the Crone lab highlighted the role of V2a INs in enhancing respiratory motor drive, demonstrating that chemogenetic stimulation improved diaphragm output for up to two weeks following SCI [91]. Romer et al. (2017) used a chemogenetic approach to demonstrate the V2a INs’ potential to activate the accessory breathing muscles [125]. Another study utilized DREADDs to silence the V2a INs in both neonatal and adult mice. The findings revealed that silencing the V2a neurons in neonatal mice altered the regularity and frequency of respiratory rhythm. In contrast, in adult mice, it not only affected the frequency of respiratory rhythm but also activated the accessory respiratory muscles [33].

Optogenetic stimulation provides another powerful tool for selectively manipulating neuronal activity with temporal precision. Selective expression of Channelrhodopsin-2 (ChR2), a photosensitive ion channel, allows for the stimulation of neurons via the administration of a light stimulus. Utilizing ChR2 expression in respiratory spinal neurons (both SpINs and PMNs), photostimulation induced rhythmic and synchronous activity in the previously inactive ipsilateral diaphragm, mirroring the contralateral hemidiaphragm after SCI. This activity persisted for one minute after the photostimulation ceased. Moreover, long-term respiratory plasticity was observed, with long and patterned intermittent photostimulation leading to a sustained recovery [126].

The combined use of chemogenetic and optogenetic approaches has further underscored the capacity of spinal INs to autonomously drive motor output, even in the absence of supraspinal input. Cregg et al. (2017) highlighted this by demonstrating that SpIN activation through both strategies could elicit PMN stimulation, thereby confirming the intrinsic motor control capabilities of spinal circuits [80]. Collectively, these findings illustrate the significant potential of chemogenetic and optogenetic neuromodulation for advancing our understanding of SpINs’ contribution to respiratory motor recovery after SCI.

Unfortunately, these technologies remain in the preclinical stage, with no known clinical trials directly utilizing DREADDs having been reported. Some optogenetics clinical trials are ongoing, but, currently, this technique is only being used to treat eye disorders [127]. However, the therapeutic potential of DREADDs for various neurological conditions is actively being explored. Future clinical trials may become viable once comprehensive safety and efficacy data are established through rigorous preclinical studies in animal models [128].

### 7.3. Transplantation of Spinal Interneurons

Aside from chemogenomic approaches, the transplantation of SpINs and neural progenitor cells is also gaining traction as a promising strategy for restoring respiratory and motor function following SCI. Stem cell transplantation, particularly using ventrally derived rat spinal cord tissue enriched with IN precursors, has demonstrated significant functional recovery of respiratory function. Studies indicate that transplanting this tissue can restore spontaneous inspiratory bursting in the phrenic nerve ipsilateral to a C2Hx [129]. In contrast, dorsally derived spinal cord tissue inhibited functional recovery, underscoring the critical role of ventrally derived INs in rehabilitation.

Neural progenitors, particularly V2a INs, have been identified as strong candidates for transplantation after SCI to enhance diaphragmatic function [52,101]. One month after their transplantation, the bilateral diaphragm amplitude in injured rats was significantly greater compared to non-transplanted controls, even under respiratory challenges such as hypercapnia and hypoxia [52]. This finding highlights the essential role of SpINs in regulating and aiding diaphragm activity during respiratory stress. The detailed protocols for generating transplantable V2a INs have been previously described by Butts et al. (2017) and Brown et al. (2014) [130,131].

Lee et al. (2014) demonstrated that fetal stem cells primed with IN progenitors develop functional IN networks within the host. These grafted progenitors responded to training stimuli such as hypoxia. Ten weeks post-C2Hx, hypoxia training resulted in a higher tidal volume in rats that received the IN progenitors than those that did not. However, electrophysiological analysis revealed that while the grafted cells exhibited spontaneous firing, their activity was not synchronized with ongoing respiratory patterns [132]. Conversely, transplants enriched with V2a progenitors showed superior outcomes, including robust diaphragmatic activity one-month post-transplant [52]. Spruance et al. (2018) expanded on this by identifying the cholinergic INs as key contributors to the integration of transplanted cells with host PMNs [133]. Additionally, they demonstrated that the application of glutamate antagonists to donor tissue altered phrenic motor output, suggesting that neurotransmitter signaling within the transplanted network plays a significant role in recovery.

More research is needed on refining transplantation protocols that ensure the precise delivery, survival, and integration of specific cell types to optimize cell replacement strategies for respiratory recovery. To promote cell integration into the host respiratory network, training protocols including physical therapy and activity-based interventions, aimed at promoting synaptic plasticity and enhancing integration, should be further explored.

### 7.4. Neurorehabilitation

SpINs play multifaceted roles in coordinating respiratory function and many other motor functions, such as locomotion [134]. Breathing and locomotion are thought to be “coupled”, with the respiratory rate increasing during physical activities, such as exercise and walking [135,136]. This increase is partly attributed to the recruitment of additional PMNs through SpIN networks [137,138]. Leveraging this coupling pathway is an emerging focus in neurorehabilitation to enhance respiratory function following SCI. Le Gal et al. (2016) demonstrated that when brainstem neurons were silenced, the locomotor drive to respiratory neurons became entirely dependent on SpINs [139]. Additionally, Sutor et al. (2022) showed that ambulatory adults with incomplete SCI and intact SpIN pathways demonstrated similar or greater locomotor–respiratory coupling compared to able-bodied adults, further highlighting the importance of this pathway in neurorehabilitation [140].

Inspiratory and expiratory muscle training can also increase the strength of inspiratory and expiratory muscles [141]. Respiratory muscle training also increases expiratory muscle strength, vital capacity, and residual volume [142]. For example, Le Foll-de Moro et al. (2005) demonstrated that wheelchair interval training improved ventilation and pulmonary function in SCI patients [143]. In another study, Terson de Paleville et al. (2013) conducted a clinical study using manually assisted treadmill stepping with body weight support, showing improvement in pulmonary function [144]. They presumed this improvement was due to the neuroplastic changes in spinal neural circuits, particularly involving SpINs. However, one participant with post-traumatic dystonia of the trunk muscles did not exhibit respiratory improvement, potentially due to disorganized SpIN circuit activity that obscured the afferent input from locomotor training. Despite this outlier, the study demonstrated an overall improvement in the average respiratory performance [144].

The evidence supports the hypothesis that locomotor–respiratory coupling via SpIN circuits can be leveraged to promote respiratory recovery after SCI. Combining respiratory muscle training with body-weight-supported stepping or interval training may amplify neuroplastic adaptations within SpIN networks, enhancing both respiratory and motor function. Future research should focus on identifying optimal training regimens that synchronize locomotor and respiratory circuits to maximize rehabilitation outcomes.

### 7.5. Respiratory Training with Hypercapnia and Intermittent Hypoxia

Hypercapnia can arise in conditions such as chronic bronchitis and emphysema, and anemias can cause hypoxia. Both hypoxia and hypercapnia may also occur due to traumatic injuries, high altitudes, or cardiovascular conditions. Under these conditions, the respiratory system must adapt to increase oxygen intake. Adult respiratory circuits often respond to both stimuli by rapidly increasing the frequency and amplitude of breaths [145].

Carie Boychuk and her team demonstrated that in bulbospinal neurons localized to the discrete region of the rostral ventral lateral medulla (RVLM), hypoxia or hypercapnia significantly but reversibly depressed the frequency of release of inhibitory neurotransmitters such as GABA. This suppression led to an increased firing rate of the normally slow-firing RVLM bulbospinal neurons, indicating that inhibitory neurotransmission plays a crucial role in modulating respiratory responses to hypercapnia and hypoxia [146]. Hypoxia strongly induces spinal neuroplasticity and may hold therapeutic potential post-injury [147,148]. Intermittent hypoxia has emerged as a promising strategy for enhancing the functional connectivity of mid-cervical SpINs. Studies have shown that AIH induces plasticity in the propriospinal network, characterized by a sustained decrease in inhibitory connections and an increase in excitatory connectivity among cervical INs [77,149]. Further, AIH has been demonstrated to increase serotonergic innervation of respiratory motor nuclei after SCI, further demonstrating the therapeutic potential of AIH intervention [150].

Sandhu et al. (2015) found that a 4 min bout of mild hypoxia altered the firing rates of 93% of neurons from C3–C4 to phrenic nerves, with approximately 40% of these exhibiting short-term potentiation [77]. These neurons were likely INs, as they were non-phrenic, synaptically connected to PMNs, fired in synchrony with inspiratory phrenic output, and had low baseline discharge rates before hypoxia. AIH combined with motor training has also been shown to upregulate spinal serotonin receptors and increase protein expression in SpINs, potentially improving motor control and coordination [149]. Successive bouts of hypoxia can cause a progressive increase in the number of excitatory connections to INs synapsing on PMNs [20]. These findings are extremely interesting, especially considering the rapid timescale on which this plasticity seems to occur, potentially reflecting the recruitment and activation of pre-existing but less active connections.

The influence of hypercapnia on the enhanced therapeutic efficacy of AIH remains uncertain. Research indicates that a poikilocapnic AIH protocol can increase motor potentials evoked by transcranial magnetic stimulation in the fingers, suggesting increased spinal motor neuron excitability either directly or indirectly via INs [151,152]. However, this enhancement was not observed in the diaphragm, indicating that the mechanisms underlying AIH-induced plasticity may vary across different motor systems [151,153].

It remains unclear whether combining acute intermittent hypercapnia (AIHc) and AIH has a compounding effect. AIH and AIHc activate distinct biochemical pathways—AIH primarily engages peripheral chemoreception, and AIHc relies on central chemoreception, leading to opposing neuromodulatory effects [154]. A recent study in healthy individuals suggests combining these approaches enhances respiratory drive but does not induce long-term facilitation [155]. It is possible that the protocols for that study were not fully optimized and, as a result, the full effect of the combined therapy was not seen. In addition to the problem of optimization, utilizing AIHc in individuals with SCI may pose additional problems as individuals with SCI often experience impaired hypercapnic responses due to chronic exposure to elevated CO_2_ levels resulting from compromised breathing function. This suggests that AIHc may not only be less effective than AIH but could also induce additional physiological distress in this population. Future studies should assess whether AIHc exacerbates respiratory distress in individuals with SCI or if specific protocols could be optimized to mitigate potential negative outcomes while maximizing therapeutic benefits. Recent clinical trials exploring AIH have demonstrated that this intervention is not only well tolerated by individuals with cSCI [156] but can increase maximal inspiratory pressure after just a single session [157]. While further investigations into AIH in a clinical setting are needed, these preliminary findings are promising for the future of AIH as respiratory therapy after cSCI.

Vose et al. (2022) outlined a comprehensive roadmap for the clinical translation of AIH, emphasizing key areas for further research and development. These include gaining a deeper mechanistic understanding of AIH-induced plasticity, optimization of treatment protocols, and exploring combination treatments to enhance neuroplasticity and functional recovery. Additionally, they stress the importance of identifying predictive biomarkers to assess patient responsiveness, the potential for benefit, ensuring AIH safety, particularly for individuals with pre-existing conditions, and developing standardized administration devices [151]. This roadmap provides a framework for advancing AIH from experimental research to clinical intervention.

### 7.6. Pharmacological Modulation of Spinal Interneurons

SpINs, influenced by various neurotransmitter systems, provide a promising avenue for therapeutic interventions aimed at restoring respiratory function after SCI. Understanding how neurotransmitter modulation affects excitatory and inhibitory SpINs can guide the development of strategies to reactivate dormant respiratory circuits. Inhibitory neurotransmission, primarily mediated via GABA and glycine, plays a crucial role in regulating respiratory networks [18,96]. Cregg et al. (2017) demonstrated that blocking GABA-A receptors with PTX and glycine receptors with STRYCH triggered rhythmic PMN activity, even after a complete C1 transection, highlighting the presence of an intrinsic cervical spinal network [80].

Serotonergic modulation also contributes significantly to spinal respiratory control. Viala et al. (1979, 1983) demonstrated that systemic administration of nialamide (a monoamine oxidase inhibitor) and DOPA induced rhythmic phrenic and hindlimb bursting in spinalized rabbits, suggesting potential interactions between locomotor and respiratory circuits, both of which are influenced by serotonin [158,159]. Similarly, Perségol and Viala (1994) demonstrated that serotonergic drugs elicited rhythmic bursting in cervical ventral roots in an in vitro neonatal rat model [160]. Combining serotonergic drugs with electrical stimulation activated previously inactive respiratory pathways. A serotonin precursor and pargyline unmasked polysynaptic connections to PMNs, enabling contralateral phrenic activation, including spontaneous tonic activity and long-latency excitations. These effects were site-dependent and were blocked by methysergide, confirming serotonin’s role [21,161].

Glutamate also plays a pivotal role in modulating SpIN activity. Lu et al. (2004) found that chemical stimulation of C1 and C2 with glutamate increased electrical activity in phrenic and intercostal nerves. Additionally, thoracic SpINs exhibited altered firing rates in response to glutamate application at C1 and C2 [162].

The dynamic interplay between excitatory and inhibitory SpINs, particularly in response to pharmacological interventions, underscores their therapeutic potential in respiratory function and recovery. Future preclinical research should focus on optimizing drug delivery and dosing, long-term efficacy and side effects, identifying biomarkers for treatment response, and conducting studies in larger animal models.

### 7.7. Glial Cells and Spinal Interneurons

SCI causes persistent glial activation alongside neuronal hyperactivity, leading to maladaptive neuroglial interactions that contribute to pathological conditions such as neuropathic pain [163]. These interactions result in the formation of abnormal synaptic circuits and dysregulated signaling pathways, exacerbating sensory and motor dysfunctions. SCI has been shown to heighten the activity of supraspinal neurons involved in pain processing, increase cervical excitatory IN activation, and reduce inhibitory IN function, creating an imbalance that fosters chronic pain. Additionally, SCI induces the activation of astrocytes in supraspinal pain-processing centers and ipsilateral ventral horn caudal to the injury site [164]. Notably, restoring glutamate transporter (GLT1) expression in cervical astrocytes has been identified as a potential therapeutic strategy. In a study by Falnikar et al. (2016), GLT1 restoration successfully reversed pre-established neuropathic pain in a cervical contusion mouse model, highlighting the role of astrocytes in modulating pain responses following SCI [165].

Following SCI, reactive astrocytes form a glial scar that limits axonal regeneration by creating both a physical barrier and a biochemically inhibitory environment. This prevents damaged axons from regenerating across the injury site, thereby hindering functional recovery. The regenerating potential of injured axons is largely determined by the distance between the axotomy site and the neuron’s cell body. SpINs, which have cell bodies located closest to the SCI site, exhibit a greater capacity for spontaneous regeneration, enabling some axons to grow through the glial scar. These regenerated SpIN axons can also evoke postsynaptic potentials in motor neurons [166].

In contrast, the corticospinal tract (CST), the primary motor pathway responsible for voluntary movements, including voluntary respiration, has cell bodies located furthest away from the injury site, making it a classic example of a poorly regenerating pathway [167,168]. The bulbospinal tract, with cell bodies positioned between SpINs and CST neurons, exhibits moderate regenerative potential. Studies have shown that implementing Schwann cell channels or nerve grafts can promote brainstem neuron regeneration after SCI, offering a promising approach for enhancing recovery [169,170,171].

The use of antagonist targeting for a central inhibitor of neuron-intrinsic axon growth potential restored respiratory function in live animals with SCI, as seen on EMG. It promoted long-distance regeneration of injured rVRG and serotonergic axons through the lesion towards PMNs spanning C3–C5 segments. Although no change in the size of the scar was seen, other possible effects, such as the stimulation of axon regeneration via the alteration of physical properties of the scar, production of growth factors, or reduction in inhibitory factors, were not analyzed and, thus, cannot be excluded. Even though synaptic input onto SpINs was not assessed, the greater amount of recovery and the small quantity of rVRG axons caudal to C3 suggest that the regenerating axons that reach the C3 are innervating INs that augment nearby PMNs and activate caudal PMNs. More preclinical assessments of overall ventilatory function, such as whole-body plethysmography or blood gas measurements, EMG recordings in response to hypercapnic, and hypoxic challenges, along with safety and efficacy analysis, need to be conducted to determine the viability of this antagonist for clinical studies [172].

### 7.8. Gene Therapy Targetting Spinal Interneurons

Gene therapy is emerging as a promising approach for modulating SpINs to facilitate recovery following SCI. One strategy involves viral gene transfer of fibroblast growth factor 22 (FGF22), a presynaptic organizer, to long propriospinal INs, excitatory INs, or broader IN populations, which can enhance specific or general circuit rewiring after incomplete SCI [173]. Likewise, targeting Gsx1, a key regulator of excitatory and inhibitory INs, has shown potential in increasing the number of neural progenitors during the acute phase following thoracic dorsal hemisection. In the chronic phase, Gsx1 expression promotes the generation of glutamatergic and cholinergic INs while reducing GABAergic INs, thereby modulating the excitatory–inhibitory balance. Additionally, Gsx1 reduces astrogliosis and scar formation, enhances serotonergic activity, and improves locomotor function in injured mice [174]. Another gene therapy approach targets brain-derived neurotrophic factor (BDNF) signaling via the tropomyosin-related kinase subtype B (TrkB) receptor in PMNs after C2Hx. Studies have shown that the viral-mediated intrapleural delivery of TrkB to PMNs is sufficient to enhance the recovery of phrenic activity after C2Hx [175,176].

Pompe disease is a lysosomal storage disease characterized by muscular dystrophy due to a deficiency in acid α-glucosidase (GAA). Among its many complications, it can lead to respiratory muscle dysfunction and failure. A promising approach to address this involves the direct intraspinal injection of viral GAA, which has been shown to induce GAA expression in motor neurons and INs at the level of the phrenic motor nucleus and beyond. This intervention significantly improved minute ventilation, as demonstrated through whole-body plethysmography [177].

While gene therapy holds great potential for enhancing respiratory function after SCI, it also presents several therapeutic challenges, including long-lasting unintended effects [178]. To develop safer and more effective gene therapies, Zeng et al. (2023) outlined future research directions, which include conducting comparative studies across different species, identifying therapeutic targets in mammals, addressing technical limitations, and integrating multiple regenerative approaches to improve patient outcomes following SCI [179].

## 8. Conclusions

An increasing body of evidence highlights the essential role of SpINs in facilitating respiratory recovery after SCI. Extensive research has examined the anatomical and functional reorganization of IN circuits following injury, revealing that specific excitatory and inhibitory subpopulations play distinct roles in shaping respiratory plasticity. Yet, several fundamental questions remain. Notably, these circuits remain dormant until injury occurs, raising intriguing possibilities about their role in other physiological processes. Could this circuitry also enhance breathing during physical exertion or in response to respiratory stressors such as hypercapnia and hypoxia? What are the underlying molecular mechanisms driving the plasticity of these circuits? These questions highlight the complexity of IN networks and suggest their broader relevance beyond injury recovery.

Delineating the mechanisms by which the SpIN circuit adapts and reorganizes after SCI will provide valuable insights into neuroplasticity. Therapeutically, the potential to harness this plasticity is tremendous. While research has traditionally focused on neuronal contributions to respiratory function, a broader exploration of non-neuronal elements, particularly glial cells, is crucial to optimizing neuroplasticity. This includes in-depth studies on astrocytes and other glial cells to identify the cellular targets most likely to improve treatment efficacy. Further investigation into the neuronal and glial interactions and functional plasticity will enable the development of more precise and effective interventions for SCI. Ultimately, the studies explored within this review, and future discoveries, may help restore respiratory motor function to near normal levels, offering hope and improved quality of life for individuals affected by SCI.

## Figures and Tables

**Table 1:** Respiratory centers in the brain and their function. Pre-BötC = Pre-Bötzinger Complex, BötC = Bötzinger Complex, PiCo = Post-inspiratory Complex, KF = Kölliker-Fuse Nucleus, pFRG = Parafacial Respiratory Group, rVRG = Rostral Ventral Respiratory Group, cVRG = Caudal Ventral Respiratory Group, LC = Locus Coeruleus, NTS = Nucleus Tractus Solitarius.

Nucleus	Function
Pre-Bötc	Inspiratory rhythm generation
Bötc	Transition between inspiration and expiration
PiCo	Post-inspiration
KF	Switch from inspiration to expiration
pFRG	Active expiration
rVRG & cVRG	Transmission of inspiratory and expiratory rhythms to PMNs
Raphe	CO_2_ chemosensation
LC	Hypercapnia sensation and response
NTS	Input acquisition from pulmonary and cardiac baro-, chemo-, and stretch receptors, as well as carotid body receptors
Hypothalamus	Physiologic modulation of respiration

**Table 2:** Summary of dorsal spinal interneuron subtypes and properties [59,60,61,62].

Dorsal Spinal Interneurons					
General Class	Class	Subtypes	Neurotransmitter	Transcription Factor	Implicated Function(s)
Class A Derive in a BMP and Wnt signaling dependent manner	dI1	X	Glutamate	Lhx2	Proprioception
	dI2	X	Glutamate	Foxd3	Proprioception, Gait
	dI3	X	Glutamate	Isl1	Grip, mechanosensation
Class B Derive in a BMP independent manner	dI4	X	GABA/Glycine	Pax2, Lbx1	Pain, itch
	dIL_A_	Nociceptin, Enkephalin, DYN, NPY, Galanin	GABA/Glycine	Pax2, Lhx1/5, Ptf1α+	Gate sensory transmission
	dI5	X	Glutamate	Lmx1b, Lbx1,	Pain, itch, heat, light touch
	dIL_B_	PACAP, GRP, SOM, NPFF, CCK, TAC1, Neurotensin, Calcitonin	Glutamate	Lmx1b, Tlx1/3	Cutaneous sensory transmission
	dI6	x	GABA/Glycine	Pax2, Dmrt3	Motor/gait

**Table 3:** Summary of ventral spinal interneuron subtypes and properties [63,64,65,66,67,68,69,70,71].

Ventral Spinal Interneurons				
Class	Subtypes	Neurotransmitter	Transcription Factor	Implicated Function(s)
V0	V0v	Glutamate	Dbx1, Evx1	Forward Trotting Locomotion, Scratching
	V0d	GABA and Glycine	Dbx1	Forward walking Locomotion, Postural correction
	V0c	Acetylcholine, Glutamate	Dbx1, Pitx2	C boutons on motor neurons, postural correction
	V0g	Glutamate	Dbx1	Unknown
V1	Extensive heterogeneity	GABA	Engrailed 1	Locomotor cycle speed
V2	V2a	Glutamate	Chx10	Left right coordination, sustain breathing after injury
	V2b	GABA	GATA3	Regulate locomotor speed
V3	x	Glutamate	Sim1	Coordinate crossed and interlimb movement, Spasticity

## Data Availability

Not applicable.
